# An African bat in Europe, *Plecotus gaisleri*: Biogeographic and ecological insights from molecular taxonomy and Species Distribution Models

**DOI:** 10.1002/ece3.6317

**Published:** 2020-04-29

**Authors:** Leonardo Ancillotto, Luciano Bosso, Sonia Smeraldo, Emiliano Mori, Giuseppe Mazza, Matthias Herkt, Andrea Galimberti, Fausto Ramazzotti, Danilo Russo

**Affiliations:** ^1^ Wildlife Research Unit Dipartimento di Agraria Università degli Studi Federico II di Napoli Portici Italy; ^2^ Dipartimento di Scienze della Vita Università degli Studi di Siena Siena Italy; ^3^ CREA Research Centre for Plant Protection and Certification Firenze Italy; ^4^ Faculty of Geo‐Information Science and Earth Observation University of Twente Enschede The Netherlands; ^5^ ZooPlantLab Dipartimento di Biotecnologie e Bioscienze Università degli Studi di Milano ‐ Bicocca Milano Italy; ^6^ School of Biological Sciences University of Bristol Bristol UK

**Keywords:** bioacoustics, biomod2, cryptic species, molecular identification, *Plecotus gaisleri*, Species Distribution Modeling

## Abstract

Because of the high risk of going unnoticed, cryptic species represent a major challenge to biodiversity assessments, and this is particularly true for taxa that include many such species, for example, bats. Long‐eared bats from the genus *Plecotus* comprise numerous cryptic species occurring in the Mediterranean Region and present complex phylogenetic relationships and often unclear distributions, particularly at the edge of their known ranges and on islands. Here, we combine Species Distribution Models (SDMs), field surveys and molecular analyses to shed light on the presence of a cryptic long‐eared bat species from North Africa, *Plecotus gaisleri*, on the islands of the Sicily Channel, providing strong evidence that this species also occurs in Europe, at least on the islands of the Western Mediterranean Sea that act as a crossroad between the Old Continent and Africa. Species Distribution Models built using African records of *P. gaisleri* and projected to the Sicily Channel Islands showed that all these islands are potentially suitable for the species. Molecular identification of *Plecotus* captured on Pantelleria, and recent data from Malta and Gozo, confirmed the species' presence on two of the islands in question. Besides confirming that *P. gaisleri* occurs on Pantelleria, haplotype network reconstructions highlighted moderate structuring between insular and continental populations of this species. Our results remark the role of Italy as a bat diversity hotspot in the Mediterranean and also highlight the need to include *P*. *gaisleri* in European faunal checklists and conservation directives, confirming the usefulness of combining different approaches to explore the presence of cryptic species outside their known ranges—a fundamental step to informing conservation.

## Graphical abstract

Here, we combine Species Distribution Models, field surveys, and molecular analyses to shed light on the long‐debated presence of a cryptic long‐eared bat species from North Africa, *Plecotus gaisleri*, on the islands of the Sicily Channel, providing strong evidence that this species also occurs in Europe, at least on the islands of Western Mediterranean that act as a crossroad between the Old Continent and Africa.

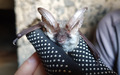

## 1 INTRODUCTION

Cryptic species are distinct biological species that are difficult or impossible to distinguish from one another due to strong morphological overlap (Bickford et al., 2007). As a result, they are often overlooked, and thus classified by taxonomists as a single nominal species (Knowlton, 1993). Such species can only be revealed as genetically isolated entities using appropriate molecular markers (Chenuil et al., 2019).

Cryptic species represent a major challenge to biodiversity assessments whenever species identification is attempted in the field solely on morphological characters (Bickford et al., 2007; Mori, Nerva, & Lovari, 2019). Ignoring the existence of cryptic species may lead to severe underestimation of species richness within a certain taxon (e.g., Funk, Caminer, & Ron, 2011), so that despite some of such species may be threatened, they are excluded from conservation actions because they remain undescribed (Delić, Trontelj, Rendoš, & Fišer, 2017). Moreover, even after cryptic species are described and thus known to science, their presence may be overlooked in field surveys, leading to underestimation of species richness, overestimation of the abundance of the nominal species (Chenuil et al., 2019), and possibly insufficient conservation (Delić et al., 2017).

The ever‐growing application of molecular techniques (favored by a continuous decrease in cost and time required for analyses) has made them fundamental in identifying cryptic species (Galimberti, Sandionigi, Bruno, Bellati, & Casiraghi, 2015). The parallel creation and growth of genetic reference data (e.g., Benson et al., 2012) have further contributed to a routine adoption of molecular tools in ecological studies.

Due to the difficulties associated with field recognition of cryptic species, their observed distributions might provide a wrong picture of their actual range. Species Distribution Models (SDMs) can help address this problem by estimating species' presence in nonsampled areas and thus inferring species' ranges (e.g., Rebelo & Jones, 2010) from a relatively limited number of known records (Guisan et al., 2006). Furthermore, SDMs represent an example of effective tools which can be applied to tackle many issues in applied ecology and support conservation planning in several ways (Bertolino et al., 2020; Maiorano, Chiaverini, Falco, & Ciucci, 2019; Mateo et al., 2019; Razgour, Rebelo, Febbraro, & Russo, 2016).

Accurate range estimates of cryptic species are clearly paramount to both biogeography and conservation biology, particularly at the edge of species' ranges (Holt & Keitt, 2005). Islands tend to house a disproportionate number of endemic species, which also raises the occurrence likelihood of cryptic species (Srinivasulu, Srinivasulu, Srinivasulu, & Jones, 2019). Detection of cryptic species on islands is hence critical and, given the high degree of isolation and scarce resources available, key to carrying out effective conservation (Conenna, Rocha, Russo, & Cabeza, 2017).

The knowledge of bat species richness in Europe has improved significantly in the last 30 years thanks to the application of integrated molecular methods, bioacoustics, and morphometric techniques, which led to recent identification of several cryptic species, for example, *Pipistrellus pygmaeus* (Barratt et al., 1997), *Eptesicus isabellinus* and *E. anatolicus* (Juste, Benda, Garcia‐Mudarra, & Ibanez, 2013), and *Myotis crypticus* (Juste, Ruedi, Puechmaille, Salicini, & Ibáñez, 2018). Palearctic long‐eared bats (genus *Plecotus*) have traditionally represented a conspicuous challenge to bat specialists, due to their complex biogeographical and phylogenetic histories, paired by a marked phenotypic convergence across most species (Ashrafi et al., 2013; Kiefer, Mayer, Kosuch, Helversen, & Veith, 2002).

*Plecotus* species occur throughout Europe, along the belt of Mediterranean climate in Northwest Africa, as well as along the Nile river valley (Benda et al., 2010; Benda, Kiefer, Hanák, & Veith, 2004). All Palearctic long‐eared bats were classified as *P. auritus* until 1960, when *P. austriacus* was formally recognized (Bauer, 1960). After this first splitting, further morphological and molecular studies evidenced a far more complex pattern of diversification within the genus across Europe and the Mediterranean basin, which led to the description of new taxa from both the *auritus* and *austriacus* clades (Juste et al., 2004; Kiefer et al., 2002; Mayer, Dietz, & Kiefer, 2007; Mucedda, Kiefer, Pidinchedda, & Veith, 2002). With the exception, within the *auritus* clade, of *P. macrobullaris* Kuzyakin, 1965, occurring in the main mountain ranges of the Western Palearctic, from the Pyrenees to the Middle East (Alberdi, Garin, Aizpurua, & Aihartza, 2013), and *P. sardus*, endemic to Sardinia (Mucedda et al., 2002), all other recently described long‐eared bats across the Mediterranean Region belong to the *austriacus* clade. Among these, *P. kolombatovici* (Dulić, 1980) is reported for the Balkans and peninsular Italy (Ancillotto et al., 2018), whereas *P. teneriffae* (Barret‐Hamilton, 1907) is restricted to three of the Canary Islands (Pestano, Brown, Suárez, Benzal, & Fajardo, 2003), and *P. christii* Gray, 1838 in Egypt, Sudan, and eastern Libya (Benda et al., 2004). *Plecotus* bats from Northwest Africa were initially assigned to *P*. *austriacus*, until molecular evidence (Benda et al., 2004; Juste et al., 2004) suggested these form a distinct clade, yet closely related to *P. teneriffae* and *P. kolombatovici*. Benda et al. (2004) described long‐eared bats from Libya as a subspecific taxon (*P. teneriffae gaisleri*) that was later recognized as a separate species, *P. gaisleri* (Benda et al., 2014), based on mitochondrial DNA divergence from congeneric taxa (Benda et al., 2004).

The range of *Plecotus gaisleri* along the coasts of northern Africa from Morocco to Libya is undisputed, but there is a debate on the occurrence of the species in Europe. The species is excluded from the current European checklist of bat species adopted within the framework of the UNEP “EUROBATS” Agreement, with the following motivation: “In Dietz and Kiefer (2016, p. 372), *P. gaisleri* is recognised as a European species, while stating ‘It is possible that this is the form that has been identified as *P. austriacus* on Pantelleria (Fichera, *in litt*.) and Malta’. In the absence of any formal publication to support this statement, the species is not accepted as occurring in Europe.” (Eurobats Meeting of Parties, 2018).

The islands that lie in the Sicily Channel represent an ideal biogeographic bridge between Africa and Europe, so a mobile species such as *P. gaisleri* might well occur there, even if potential interspecific competition with European *Plecotus* populations may limit its spreading further north, to mainland Europe. Very recently, two studies (Batsleer et al., 2019; Mifsud & Vella, 2019) established with molecular markers that *P. gaisleri* does occur on Malta, while its presence on Pantelleria remained an open question. Long‐eared bats from Pantelleria have been recorded and collected in the past (Felten & Storch, 1970;Zava & Lo Valvo, 1990) and were identified as *P. austriacus*. Their preserved skulls were subsequently used for morphometric analyses that evidenced their distinctiveness when compared to skulls from across Europe, while they clustered along with specimens from North Africa (Spitzenberger, Strelkov, Winkler, & Haring, 2006). Based on cranial morphology, Spitzenberger et al. (2006) also hypothesized that *P. gaisleri* and *P. kolombatovici* may occur in sympatry on Pantelleria, yet only one out of six skulls examined potentially belonged to the latter taxon. Other authors considered the co‐occurrence of the two species on Pantelleria unlikely (Dietz & Kiefer, 2016; Lanza, 2012), and the island was also classified as unsuitable for *P. kolombatovici* by recent modeling work (Ancillotto, Mori, Bosso, Agnelli, & Russo, 2019).

**Figure 1:**
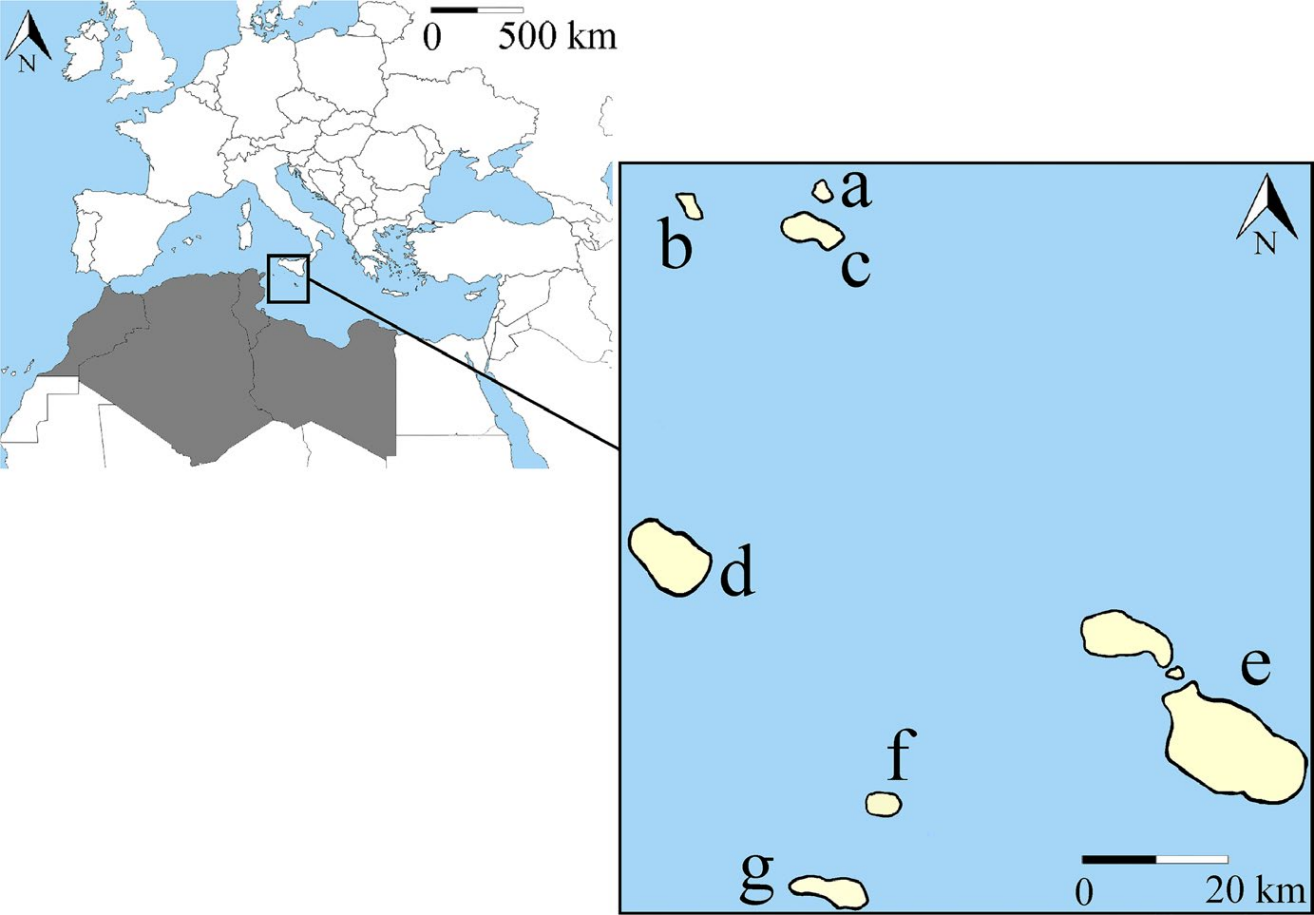
Study area considered to model *Plecotus gaisleri* potential distribution. Dark gray shows the species' known/potential geographic range (training area), while in the zoomed frame, the projection area is shown in light yellow. The islands are labeled as follows: Levanzo (a), Marettimo (b), Favignana (c), Pantelleria (d), Malta and Gozo (e), Linosa (f), and Lampedusa (g). Distances among islands were slightly modified to include all islands in the image.

On such grounds, we used a combination of genetic multilocus analysis, field surveys, and spatial modeling to test the hypothesis that *P. gaisleri* occurs in southern Europe somewhere else besides Malta. More specifically, we expect its presence across other Sicily Channel Islands because of their position between the African and European bioregions (Figure 1), and their environmental conditions closely resembling those of coastal North Africa. These islands are in fact strong candidates for the presence of this species. Moreover, long‐eared bats from the *austriacus* group are frequently reported on islands (Pestano et al., 2003), including some in the Sicily Channel (Felten & Storch, 1970; Mifsud & Vella, 2019). To test our hypothesis, we first built an SDM for *P. gaisleri* from North Africa, projecting it to the islands found in the Sicily Channel to assess their potential environmental suitability. We then carried out ad hoc field surveys considering SDM results. To validate our modeling exercise, we used known records from Pantelleria and Malta and new records obtained through molecular identification of specimens from Pantelleria examined for the present study. DNA sequence data also allowed us to assess the genetic relationships between bats from the Sicily Channel and Palearctic long‐eared bats.

## 2 MATERIALS AND METHODS

### 2.1 Species Distribution Modeling

#### 2.1.1 Area of training and projection

Our training area comprised the entire territories of Morocco, Algeria, Tunisia, and Libya between latitudes 37°N–18°N and longitudes −14°W to 25°E (corresponding to ca. 4,752,160 km^2^) (Figure 1). The model projection area included the following islands located between North Africa and Sicily (Southern Italy): Levanzo, Marettimo, Favignana, Pantelleria, Malta and Gozo, Linosa, and Lampedusa (Figure 1). The projection area ranged between latitudes 38°N–35°N and longitudes 11°E–14°E (corresponding to ca. 462 km^2^).

#### 2.1.2 Presence records of *Plecotus gaisleri*

We built the SDM for *P. gaisleri* using records collected by Herkt, Barnikel, Skidmore, and Fahr (2016) and the online database iNaturalist (section African bats—www.inaturalist.org/projects/afribats). We only used records situated in the geographic training area resulting in 25 records of *P. gaisleri*. These were screened in ArcGIS (version 10.2.2) for spatial autocorrelation using average nearest neighbor analyses to remove spatially correlated data points (e.g., Bauder, Stevenson, Sutherland, & Jenkins, 2017; Bosso et al., 2018; Kwon, Kim, & Jang, 2016; Mohammadi, Ebrahimi, Moghadam, & Bosso, 2019). The process provided 17 independent, quality‐checked presence records to generate the SDM.

#### 2.1.3 Ecogeographical variables

To build a SDM for *P. gaisleri*, we started from a set of 21 ecogeographical variables. These included 19 bioclimatic variables plus elevation obtained from the WorldClim database ver. 2.0. (www.worldclim.org/current) (Fick & Hijmans, 2017), as well as from the global landcover map (ver. 2.0.7; http://maps.elie.ucl.ac.be/CCI/viewer) recently developed by the European Space Agency. These bioclimatic variables are derived from monthly temperature and rainfall values to generate more biologically meaningful variables. They represent annual trends (e.g., mean annual temperature, annual precipitation), seasonality (e.g., annual range in temperature and precipitation), and extreme or limiting environmental factors (e.g., temperature of the coldest and warmest month, and precipitation of the wet and dry quarters). A quarter is a period of three months (1/4 of the year) (Hijmans, Cameron, Parra, Jones, & Jarvis, 2005). Elevation is a topographical variable that represents a location's height above sea level, while the CLC is a vector map composed of an inventory of land cover classes divided into homogeneous landscape units. The elevation and bioclimatic variables represent continuous, ratio‐scaled data, while CLC variables are categorical, discrete ones. We downloaded the bioclimatic variables in GeoTiff format (.tif) choosing the 30‐arc second resolution (this corresponds to a pixel size of 0.93 × 0.93 km = 0.86 km^2^ at the equator). We clipped the variables on the area of training and projection using the “*clip*” tool in ArcGIS (ver. 10.2.2) and converted them in ASCII files using SDMtoolbox (Ver. 2.2) (Brown, Bennett, & French, 2017). After resampling all ecogeographical variables to a resolution of ca. 1 km^2^, we generated Pearson's correlation matrix with SDMtoolbox (ver. 2.2) in ArcGIS (ver. 10.2.2) and removed all highly correlated variables, retaining only variable pairs with *r* < .70 (e.g., Ancillotto et al., 2018; Niemuth et al., 2017). This led to a final set of eight ecogeographical variables used for model training: elevation (m), land cover (category—for further details, see Table S1), isothermality (%), temperature annual range (°C), mean temperature of driest quarter (°C), mean temperature of coldest quarter (°C), precipitation seasonality (%), and precipitation of coldest quarter (mm).

#### 2.1.4 Species Distribution Models

We built the SDM using an ensemble forecasting approach, as implemented in the R package “*biomod2*” (https://cran.r‐project.org/bin/windows/base/; Thuiller, Lafourcade, Engler, & Araújo, 2009). We considered five modeling techniques (Thuiller et al., 2009): (a) maximum entropy models (MAXENT); (b) generalized linear models (GLM); (c) generalized additive models (GAM); (d) generalized boosted models (GBM); and (e) random forests (RF; for further details, see Thuiller et al., 2009). In agreement with previous studies (e.g., Smeraldo et al., 2020; Tulowiecki, 2020), GLMs and GAMs were calibrated using a binomial distribution and a logistic link function, while GBMs were developed with the maximum number of trees set to 5,000, threefold cross‐validation procedures to select the optimal number of trees to be kept, and a value of seven as maximum depth of variable interactions. Random forest models were fitted by growing 750 trees with half the numbers of available predictors sampled for splitting at each node. MAXENT models were fitted with default settings and a maximum value of 1,000 iterations. To avoid model overfitting, we developed MAXENT models applying species‐specific settings selected using the “ENMeval” (e.g., Fourcade, Besnard, & Secondi, 2018) R package. This approach runs successively several MAXENT models using different combinations of parameters to select the settings that optimize the trade‐off between goodness of fit and overfitting. We set ENMeval to test regularization values between 0.5 and 4, with 0.5 steps, as well as the following feature classes: linear, linear + quadratic, hinge, linear + quadratic + hinge, linear + quadratic + hinge + product, and linear + quadratic + hinge + product + threshold, which correspond to the default ENMeval settings. We then selected the parameters that scored lower AIC values.

We calibrated our models in a training area including Morocco, Algeria, Tunisia, and Libya, and projected them to Levanzo, Marettimo, Favignana, Pantelleria, Malta and Gozo, Linosa, and Lampedusa. The occurrence dataset was randomly split into a 70% sample, used for the calibration of the model, and the remaining 30%, used to evaluate model performance. Because our dataset contained only occurrence data, a set of 10,000 background points were randomly placed over the training area. The data splitting procedure was repeated 10 times and the evaluation values averaged. We ran a total of 50 SDMs (five algorithms × 10 splitting replicates for model evaluation) that were then projected over the study area. The relative importance of variables was also calculated from the ensemble model using the specifically devoted functionality available in the biomod2 package (Jiguet, Barbet‐Massin, & Henry, 2010). The final potential distribution was obtained by averaging the projections from the 10 replicated ensemble models generated through the subsampling procedure (see above). The average final map obtained had a logistic output format with suitability values from 0 (unsuitable habitat) to 1 (suitable habitat). The final map was then binarized into presence–absence values using a threshold that maximizes sensitivity (the percentage of correctly predicted presence) and specificity (the percentage of correctly predicted absence; Fielding & Bell, 1997). This threshold has been widely used (e.g., Algar, Kharouba, Young, & Kerr, 2009; Dubuis et al., 2011; Smeraldo et al., 2020) and is among the most accurate ones (Liu, Berry, Dawson, & Pearson, 2005).

To avoid major model uncertainty, variables in the projection area must meet a condition of environmental similarity with the environmental data used for calibrating the model. Therefore, we first ascertained that this condition occurred by inspecting the multivariate environmental similarity surfaces (MESS) generated by Maxent (e.g., Archis, Akcali, Stuart, Kikuchi, & Chuncom, 2018; Jarnevich et al., 2018).

#### 2.1.5 Model validation

Predictive performances of SDMs were assessed by measuring the area under the receiver operating characteristic curve (AUC; Hanley & McNeil, 1982) and the true skill statistic (TSS; Allouche, Tsoar, & Kadmon, 2006). These validation methods have been widely used and found to perform well (Breiner, Guisan, Bergamini, & Nobis, 2015; Mohammadi et al., 2019; Smeraldo et al., 2018). After excluding models with AUC < 0.7, model averaging was performed by weighting the individual model projections by their AUC scores, a method shown to be particularly robust (Marmion, Parviainen, Luoto, Heikkinen, & Thuiller, 2009). Finally, to validate our models we used all presence records of *Plecotus* bats collected in the projection area in this study or in past published surveys (Table S2), and identified as *P. gaisleri* (Batsleer et al., 2019; Mifsud & Vella, 2019). All records were overlapped to logistic and binary maps of *P. gaisleri* in ArcGis (ver. 10.2.2), and then, for each point we extracted the pixel value of the maps using the tool “Extract value to point.”

### 2.2 Field validation

#### 2.2.1 Study area

Fieldwork was carried out in September 2019 within the territory of the Pantelleria National Park (36°47′06″N, 11°59′30″E) on Pantelleria Island. The latter is a volcanic island of ca. 80 km^2^ located in the middle of the Sicily Channel, ca.70 and 100 km off the African and Sicilian coasts, respectively. Apart from Malta, Pantelleria is the only island in the Sicily Channel for which long‐eared bat records are available; hence, it provides an ideal set to test the validity of the SDM and search for material for molecular identification. The island has a typical Mediterranean climate with a mean annual precipitation of 409 mm, concentrated in autumn and early spring, and a mean monthly temperature ranging between 11.7 and 25.6°C (Gianguzzi, 1999). Mediterranean scrubland dominates the natural vegetation on the island, which also comprises large portions of bare volcanic rocks and cultivated patches (mainly vines and capers), part of which abandoned. Mediterranean woodlands made of conifers (mostly *Pinus pinaster* Aiton) and oaks (*Quercus ilex* L.) are concentrated in the mountainous sections of the central and southern part of the island (maximum altitude: 815 m a.s.l.). One large brackish water lake is present on the island, whereas freshwater only occurs in one artificial permanent reservoir and, in early spring and autumn, in a few temporary ponds.

#### 2.2.2 Bat sampling

We assessed the presence of long‐eared bats on the island by combining acoustic surveys, roost inspections, and temporary capture of bats. Selection of sampling sites was first aided by the SDM outputs we used to locate highly suitable sites, followed by on‐ground surveys that also relied on in situ habitat assessment. From sunset to dawn, we used automatic D500x detectors (Pettersson Elektronik AB) placed opportunistically across the island's potentially suitable area. We recorded bat activity at eight sites, equally distributed in four habitat types: water habitats, urban/rural interface, Mediterranean scrubland, and woodland. Sites were at least 500 m apart from each other (mean ± *SD*: 861.6 ± 265.5 m), and recordings were made once at each site. Recordings were then visually inspected and main call variables manually measured in BatSound v3.31 (Pettersson Elektronik). No bats from other genera present on Pantelleria emit echolocation calls resembling those of *Plecotus* sp. We refrained from attempting species identification because echolocation call structure among *Plecotus* species shows considerable overlap. Instead, we only examined recordings to identify calls at the genus level: *Plecotus* sp. calls have a relatively steep FM spectrogram and are characterized by a prominent second harmonic, so identification at that level is reliable (Russo & Jones, 2002). We used acoustic data to assess long‐eared bat distribution on the island and support further activities such as capture and roost inspection.

We explored potential roosts such as mines, tunnels, abandoned rural buildings, and caves, searching for bats or their signs of presence (droppings, urine stain on walls, prey remains) which we located by consulting published sources (Felten & Storch, 1970), as well as following the advice of islanders and park authorities. Mistnets were mounted over watersites, along potential flight corridors in woodland and near potential roosts. We identified captured bats visually, established their sex, age, and reproductive status and measured forearm length, tragus size, and body mass with a digital caliper and a scale, respectively. A 3‐mm biopsy punch was also taken from wing membranes and immediately stored in 99% ethanol for subsequent DNA‐based species identification (Galimberti et al., 2012). Bats were released soon after capture, and no voucher was taken (Russo, Ancillotto, Hughes, Galimberti, & Mori, 2017).

#### 2.2.3 DNA‐based identification

Total DNA was extracted from the *Plecotus* tissue samples collected on Pantelleria by using the DNeasy Blood & Tissue Kit (Qiagen) following manufacturer's instructions. Purified DNA concentration and quality of the samples were estimated fluorometrically with a NanoDrop™ 1000 Spectrophotometer (Thermo Scientific). To confirm the putative species identification, genetic regions from the three mitochondrial loci COI (658 bp), ND1 (1,385 bp), and 16s rRNA (548 bp) were amplified and sequenced. These loci were chosen because of their reliability in distinguishing echolocating bat species (including those belonging to the genus *Plecotus*, see Mayer et al., 2007; Benda et al., 2004; Galimberti et al., 2012) and due to the large abundance of reference sequences in accessible databases (i.e., GenBank https://www.ncbi.nlm.nih.gov/ and BOLD http://www.boldsystems.org/) serving as comparison for our case study. The three loci were amplified and sequenced as described in Galimberti et al. (2012) (COI), Mayer and von Helversen (2001) (ND1), and Mucedda et al. (2002) (16s rRNA). After sequencing, primer nucleotide sequences trimming, and sequences quality check, the presence of stop codons was verified by using the online tool EMBOSS Transeq (http://www.ebi.ac.uk/Tools/st/emboss_transeq/). Sequence data were submitted to the European Bioinformatics Institute of the European Molecular Biology Laboratory (EMBL‐EBI) and assigned to the accession numbers provided in Table 1. To assign taxonomically the *Plecotus* bats sampled on Pantelleria, the obtained sequences were first queried against the GenBank (BLAST algorithm) and the BOLD (IDS tool) databases for the three loci and the COI only, respectively. Second, to assess the genetic divergence among these specimens and other *Plecotus* species, we assembled multiple alignments (one for each locus) including the sequences obtained in this study and the available sequences from GenBank and BOLD (Table 1). Given the already known affinity of *Plecotus* from Pantelleria with representatives of the *“austriacus”* group (i.e., *P. austriacus, P. kolombatovici*, *P. christii*, *P. teneriffae,* and *P. gaisleri*), we decided to consider only these species in the analysis. Multiple sequence alignments were produced using MAFFT online (https://mafft.cbrc.jp/alignment/server/ Katoh, Asimenos, & Toh, 2009) with default parameters. Due to different lengths of available sequences, each alignment was trimmed to the same final length. Genetic “uncorrected *p*‐distances” were calculated by using MEGA 7 (Kumar, Stecher, & Tamura, 2016). Finally, to investigate whether sampled bats clustered with currently known geographic lineages of *Plecotus*, the mitochondrial haplotypes of the Pantelleria population were examined at each locus using a haplotype network reconstruction. The number of haplotypes was calculated with DnaSP v6 (Rozas et al., 2017), and the unrooted minimum spanning networks were obtained using the median‐joining algorithm (Bandelt, Forster, & Röhl, 1999) implemented in PopART (http://popart.otago.ac.nz/howtocite.shtml—default settings) (Leigh & Bryant, 2015).

**Table 1:** Multilocus molecular dataset of the *Plecotus* “*austriacus”* group

Taxon	Sample name	16s		COI		ND1		Country/Locality
		a.n.	H	a.n.	H	a.n.	H	
*P. austriacus*	MHNG1806.042	_	_	ABBWP036‐06	B1	_	_	Switzerland
*P. austriacus*	MHNG1806.050	_	_	ABBWP039‐06	B1	_	_	Switzerland
*P. austriacus*	MHNG1807.029	_	_	ABBWP042‐06	B1	_	_	Greece
*P. austriacus*	MHNG1807.030	_	_	ABBWP043‐06	B1	_	_	Greece
*P. austriacus*	NMP49045	_	_	ABBWP196‐07	B1	_	_	Greece
*P. austriacus*	NMP50440	_	_	ABBWP199‐07	B1	_	_	Bulgaria
*P. austriacus*	pb2441	_	_	ABBWP221‐07	B1	_	_	Slovakia
*P. austriacus*	pb2437	_	_	ABBWP266‐07	B1	_	_	Slovakia
*P. austriacus*	MIBZPL01252	_	_	FR856811	B2	_	_	Italy
*P. austriacus*	MIBZPL01497	_	_	FR856812	B3	_	_	Italy
*P. austriacus*	Paus1389	_	_	_	_	AF401366	C6	Germany
*P. austriacus*	Paus3333_I	_	_	_	_	DQ915065	C7	Italy
*P. austriacus*	Paus4206_GR	_	_	_	_	DQ915066	C8	Greece
*P. austriacus*	Paus4212_GR	_	_	_	_	DQ915067	C9	Greece
*P. austriacus*	PausSar11	AY175820	A10	_	_	_	_	Italy_Sardinia
*P. austriacus*	PausSar9	AY175823	A11	_	_	_	_	Italy_Sardinia
*P. austriacus*	Pleaus2	DQ294111	A13	_	_	_	_	Austria
*P. austriacus*	Paus1373	AY134022	A2	_	_	AF401367	C3	Germany
*P. austriacus*	IZEA 322	AF326107	A2	_	_	_	_	Switzerland
*P. austriacus*	MHNG 3000.002	MF423097	A2	_	_	_	_	Switzerland
*P. austriacus*	_	AJ431659	A3	_	_	_	_	Spain
*P. austriacus*	3217Paus	AY134023	A4	_	_	AF516270	C4	Spain
*P. austriacus*	3209Paus	AY134024	A5	_	_	AF516271	C5	Spain
*P. austriacus*	PausSar12	AY175814	A6	_	_	_	_	Italy_Sardinia
*P. austriacus*	PausSar10	AY175815	A7	_	_	_	_	Italy_Sardinia
*P. austriacus*	PausSar3	AY175816	A8	_	_	_	_	Italy_Sardinia
*P. austriacus*	PausSar6	AY175817	A9	_	_	_	_	Italy_Sardinia
P. austriacus*	P8	LR742726	A12	_	_	_	_	Italy
*P. cf gaisleri*	MHNG1806.051	_	_	ABBWP040‐06	B9	_	_	Datasw
*P. cf gaisleri*	Ple27933	DQ294127	A23	_	_	_	_	Morocco
*P. cf gaisleri*	Pindet4	AY531620	A27	_	_	_	_	Morocco
*P. cf gaisleri*	Pindet5	AY531622	A29	_	_	_	_	Morocco
*P. cf gaisleri*	Pindet3	AY531623	A30	_	_	_	_	Morocco
*P. cf gaisleri*	MHNG 1806.051	GU328043	A31	_	_	_	_	Morocco
*P. christii*	NMP49862	_	_	ABBWP263‐07	B4	_	_	Libya
*P. christii*	NMP49863	_	_	ABBWP275‐07	B4	_	_	Libya
*P. christii*	NMP90496	_	_	ABBWP318‐07	B5	_	_	Egypt
*P. christii*	NMP90497	_	_	ABBWP330‐07	B6	_	_	Egypt
*P. christii*	Pchr	AY531615	A15	_	_	_	_	Libya
*P. gaisleri*	NMP48330	_	_	ABBWP163‐06	B10	_	_	Libya
*P. gaisleri*	NMP48331	_	_	ABBWP164‐06	B10	_	_	Libya
*P. gaisleri*	Pcfgai5055_L	_	_	_	_	DQ915064	C16	Libya
*P. gaisleri*	Pl01	MN028699	A1	MN031800	B11	MN158256	C1	Malta
*P. gaisleri*	Pl02	MN028700	A1	MN031801	B11	MN158257	C1	Malta
*P. gaisleri*	Pl03	MN028701	A1	MN031802	B11	MN158258	C1	Malta
*P. gaisleri*	Pl04	MN028702	A1	MN031803	B11	MN158259	C1	Malta
*P. gaisleri*	Pl05	MN028703	A1	MN031804	B11	MN158260	C1	Malta
*P. gaisleri*	Pl06	MN028704	A1	MN031805	B11	MN158261	C1	Malta
*P. gaisleri*	Pl07	MN028705	A1	MN031806	B11	MN158262	C1	Malta
*P. gaisleri*	Pl08	MN028706	A1	MN031807	B12	MN158263	C1	Malta
*P. gaisleri*	Pl09	MN028707	A1	MN031808	B11	MN158264	C1	Malta
*P. gaisleri*	Pl10	MN028708	A1	MN031809	B11	MN158265	C1	Malta
*P. gaisleri*	Pl11	MN028709	A1	MN031810	B11	MN158266	C1	Malta
*P. gaisleri*	Pl12	MN028710	A1	MN031811	B11	MN158267	C1	Malta
*P. gaisleri*	Pl13	MN028711	A1	MN031812	B11	MN158268	C2	Malta
*P. gaisleri*	Pl14	MN028712	A1	MN031813	B11	MN158269	C1	Malta
*P. gaisleri*	Pl15	MN028713	A1	MN031814	B11	MN158270	C1	Malta
*P. gaisleri*	Pl16	MN028714	A1	MN031815	B11	MN158271	C1	Malta
*P. gaisleri*	Pl17	MN028715	A1	MN031816	B11	MN158272	C1	Malta
*P. gaisleri*	Pl18	MN028716	A1	MN031817	B11	MN158273	C1	Malta
*P. gaisleri*	Pl19	MN028717	A1	MN031818	B11	_	_	Malta
*P. gaisleri*	Pl20	MN028718	A1	MN031819	B11	MN158274	C1	Malta
*P. gaisleri*	Pl21	MN028719	A1	MN031820	B11	_	_	Malta
*P. gaisleri*	Pl22	MN028720	A1	MN031821	B11	MN158275	C1	Malta
*P. gaisleri*	Pl23	MN028721	A1	MN031822	B11	MN158276	C1	Malta
*P. gaisleri*	Pl24	MN028722	A1	MN031823	B11	MN158277	C1	Malta
*P. gaisleri*	Pl25	MN028723	A1	MN031824	B11	MN158278	C1	Malta
*P. gaisleri*	Pl26	MN028724	A1	MN031825	B11	MN158279	C1	Malta
*P. gaisleri*	Pl27	MN028725	A1	MN031826	B11	MN158280	C1	Malta
*P. gaisleri*	Pl28	MN028726	A1	MN031827	B11	MN158281	C1	Malta
*P. gaisleri*	Pl29	MN028727	A1	MN031828	B11	MN158282	C1	Malta
P. gaisleri*	P2	LR742724	A1	LR742722	B11	LR742720	C1	Italy_Pantelleria
P. gaisleri*	P3	LR742725	A1	LR742723	B11	LR742721	C1	Italy_Pantelleria
*P. gaisleri*	Pindet2	AY531624	A1	_	_	_	_	Libya
*P. gaisleri*	Pindet1	AY531621	A28	_	_	_	_	Libya
*P. kolombatovici*	NMP48725	_	_	ABBWP176‐06	B7	_	_	Greece
*P. kolombatovici*	NMP48726	_	_	ABBWP177‐06	B7	_	_	Greece
*P. kolombatovici*	NMP90398	_	_	ABBWP304‐07	B8	_	_	Cyprus
*P. kolombatovici*	Pausk2127	_	_	_	_	AF401362	C10	Croatia
*P. kolombatovici*	Pausk1874	_	_	_	_	AF401364	C12	Greece
*P. kolombatovici*	TR1	_	_	_	_	KF218569	C14	Turkey
*P. kolombatovici*	TR2	_	_	_	_	KF218570	C15	Turkey
*P. kolombatovici*	Pkol1	AY134025	A14	_	_	AF401363	C11	Croatia
*P. kolombatovici*	Pausk1868	AY134026	A14	_	_	AF401365	C13	Greece
*P. kolombatovici*	Pkol4	AY531616	A16	_	_	_	_	Turkey
*P. kolombatovici*	Pkol3	AY531617	A17	_	_	_	_	Turkey
*P. kolombatovici*	Pkol5	AY531618	A18	_	_	_	_	Turkey
*P. kolombatovici*	Pkol6	AY531619	A19	_	_	_	_	Greece
*P. kolombatovici*	Ple202	DQ294107	A20	_	_	_	_	Libya
*P. kolombatovici*	Plekol1	DQ294109	A21	_	_	_	_	Croatia
*P. kolombatovici*	PlespTR	DQ294110	A22	_	_	_	_	Turkey
*P. teneriffae*	_	AJ431654	A24	_	_	_	_	Spain_Canary Islands
*P. teneriffae*	_	AJ431655	A24	_	_	_	_	Spain_Canary Islands
*P. teneriffae*	_	AJ431656	A25	_	_	_	_	Spain_Canary Islands
*P. teneriffae*	_	AJ431657	A26	_	_	_	_	Spain_Canary Islands
*P. teneriffae*	_	AJ431658	A26	_	_	_	_	Spain_Canary Islands

Note

Haplotype and sampling details for each considered sequence are reported. Samples genotyped in this study are marked with an asterisk.

To interpret these results, we adopted the most widely accepted nomenclature for the Afro‐Mediterranean *Plecotus* species, considering *P. gaisleri* as a distinct species, i.e. separated from *P. teneriffae* (Benda et al., 2014; Juste et al., 2004; Mayer et al., 2007); Maghrebian long‐eared bats from Morocco are currently classified as *P. gaisleri*, but morphological and molecular evidences both indicate the distinctiveness of this clade (Spitzenberger et al., 2006). Therefore, in our analysis we indicated Maghrebian bats as “*P*. cf. *gaisleri.”*

## 3 RESULTS

### 3.1 Potential distribution of *Plecotus gaisleri*

**Figure 2:**
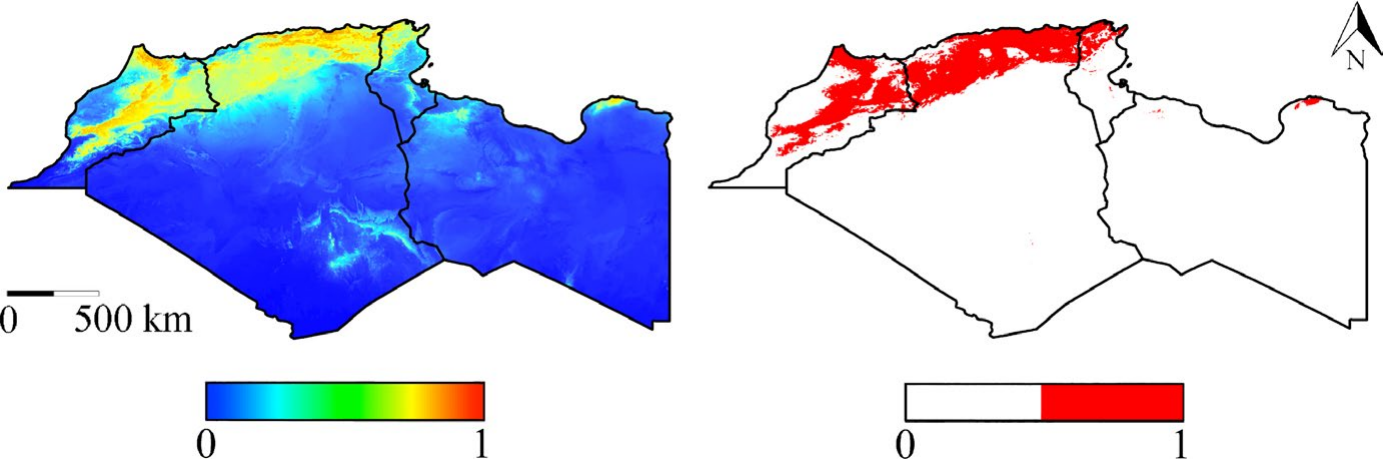
*Plecotus gaisleri* Species Distribution Models in the training areas (from left to right, Morocco, Algeria, Tunisia, and Libya). Left: logistic map; right: binary map. Scales show the probability of presence ranging from 0 to 1

**Figure 3:**
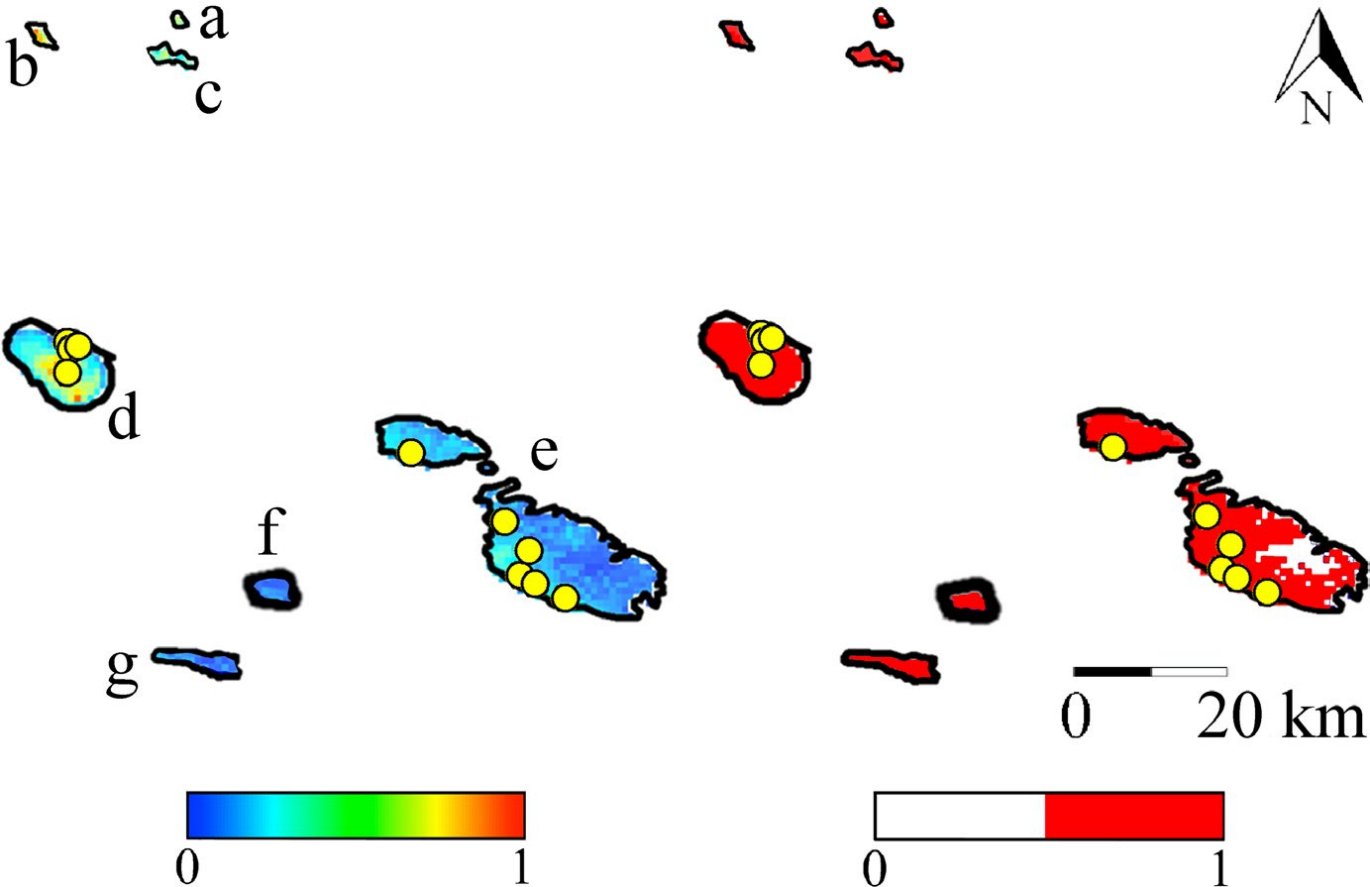
Species Distribution Models of African *Plecotus gaisleri* projected onto the Sicily Channel archipelagos. The islands are labeled as follows: Levanzo (a), Marettimo (b), Favignana (c), Pantelleria (d), Malta and Gozo (e), Linosa (f), and Lampedusa (g). Left: logistic map; right: binary map. Scales show the probability of presence ranging from 0 to 1. Yellow circle = presence records of *P. gaisleri* used for model validation (for further details, see Table S2). Distances among islands were modified for clarity

The analysis of single bioclimatic variable contributions showed that mean temperature of the coldest quarter, isothermality, and precipitation of the coldest quarter were the main ecogeographical variables influencing model performance. Based on model predictions, *P. gaisleri* showed a higher probability of occurrence where mean temperature of the coldest quarter is <10°C, isothermality <35%, and mean precipitation of coldest quarter is >50 mm, at sites dominated by typical Mediterranean forest, scrubland, and mosaic natural vegetation (Figure S1). Moreover, the probability of presence gradually decreased for higher altitudes, in particular >1,000 m a.s.l. (Figure S1). In the training area, the model predicted a high probability of *P. gaisleri* presence primarily in the mountainous parts of Morocco, northern Algeria, Tunisia, and Libya (Figure 2), yet partly extending to coastal lowlands. In the projection areas, our models predicted a medium to high probability of *P. gaisleri* presence on all islands of the Sicily Channel (Figure 3).

The analysis of multivariate environmental similarity surfaces showed that the projection area had a medium to high (from 0.67 to 1.9) environmental similarity with most of the training area (Figure S2) and that Malta, Gozo, Pantelleria, and Favignana had the highest environmental similarity of all islands.

Species Distribution Models showed excellent predictive performances as indicated by AUC and TSS, which had a mean value ± standard deviation, respectively, of 0.98 ± 0.03 and 0.90 ± 0.05. All occurrences of *P. gaisleri* used for model validation fell within potentially highly suitable areas, with logistic values between 0.6 and 0.9 (0.7 ± 0.07), all corresponding to a binary value of 1 (Table S2).

### 3.2 *Plecotus* bats on Pantelleria

**Figure 4:**
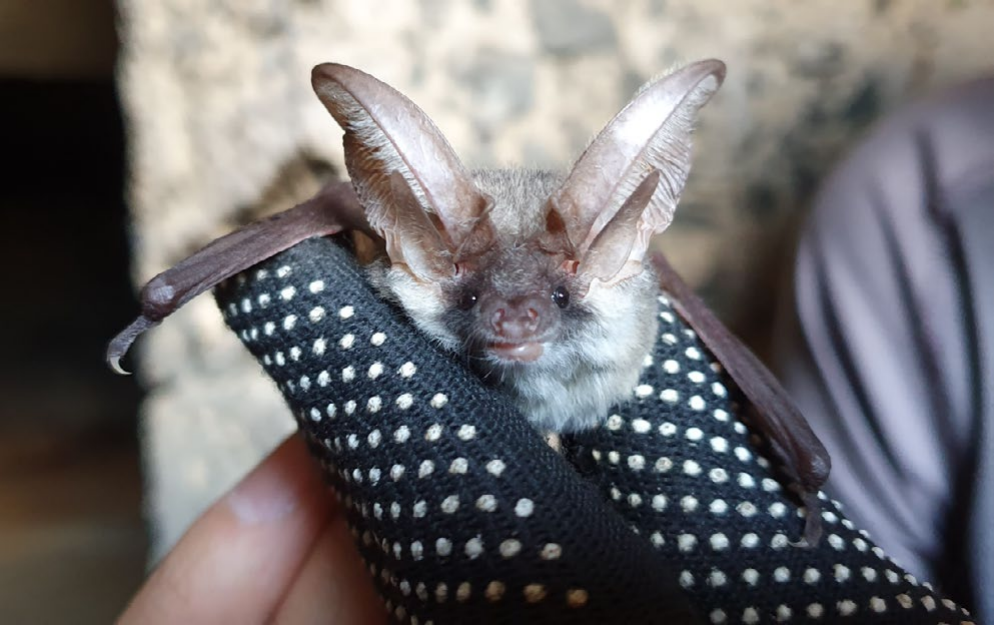
Adult male *Plecotus gaisleri* captured on the island of Pantelleria

We inspected 24 potential roosts scattered across the island (3 artificial tunnels, 6 natural caves, and 15 abandoned buildings). We found evidence of the presence of long‐eared bats at one roost and at four recording sites. We captured two adult male long‐eared bats (Figure 4; (body weight: 7.1–8.4 g; forearm length: 38.5–38.8 mm; thumb length: 5.6–6.6 mm; tragus length: 15.6–14.5 mm; tragus width: 5.0–4.8 mm) near a roost site in a tunnel. We recorded calls of *Plecotus* bats at two water sites, plus at one woodland and one scrubland site, all in the same area of the island, and recorded *Plecotus* calls quite frequently (70% of recorded passes) at one freshwater site. Echolocation calls (*n* = 51) had a frequency of maximum energy (mean ± *SD*) = 32.3 ± 2.7 kHz, start frequency = 45.7 ± 1.4, end frequency = 21.5 ± 1.0 kHz, and duration = 2.8 ± 1.0 ms.

### 3.3 DNA‐based identification

All three mitochondrial genes fragments were successfully sequenced for the two *Plecotus* samples from Pantelleria, and no stop codons were found. The two sampled bats shared the same haplotype at each locus (Table 1) and (for COI only) the BLAST search returned a 100% maximum identity match with *P. gaisleri* in all cases (i.e., COI, ND1, and 16s rRNA, query coverage 100%). The same result was obtained using the BOLD–IDS tool for the DNA barcode COI locus.

The three multiple alignments encompassing all publicly available sequences of the “*austriacus”* group contained neither stop codons nor *indels* (in the case of the COI and ND1), and after trimming, alignments were 556 bp (COI), 800 bp (ND1), and 516 bp (16s rRNA) long (see Table 1 for the composition of the three multiple alignments).

P‐distance values confirmed the marked genetic divergence of *P. gaisleri* (from Pantelleria, Malta, and Libya) from the other *Plecotus* species belonging to the “*austriacus”* group, including *P.* cf. *gaisleri* from Morocco and *P. teneriffae* from Canary Islands (Table 2).

**Table 2:** Values of genetic p‐distance divergence among *Plecotus* species (and lineages)

Lineage I	Lineage II	COI	ND1	16s rRNA
		p‐dist (S.E)	p‐dist (S.E)	p‐dist (S.E)
*P. austriacus*	*P. kolombatovici* (Balkans)	0.0987 (0.0115)	0.1175 (0.0112)	0.0551 (0.0097)
*P. austriacus*	*P. christii*	0.1275 (0.0134)	_	0.0678 (0.0108)
*P. austriacus*	*P. kolombatovici* (Turkey‐Libya)	_	0.1272 (0.0116)	0.0479 (0.0087)
*P. austriacus*	*P. *cf. *gaisleri*	0.1023 (0.0127)	_	0.0471 (0.0085)
*P. austriacus*	*P. teneriffae*	_	_	0.0523 (0.0091)
*P*. cf. *gaisleri*	*P. teneriffae*	_	_	0.0196 (0.0052)
*P. christii*	*P. kolombatovici* (Turkey‐Libya)	_	_	0.0562 (0.0098)
*P. christii*	*P.* cf. *gaisleri*	0.1125 (0.0126)	_	0.0481 (0.0090)
*P. christii*	*P. teneriffae*	_	_	0.0438 (0.0086)
*P. gaisleri*	*P. austriacus*	0.0960 (0.0119)	0.1238 (0.0119)	0.0478 (0.0089)
*P. gaisleri*	*P. kolombatovici (Balkans)*	0.0500 (0.0080)	0.0638 (0.0082)	0.0230 (0.0062)
*P. gaisleri*	*P. christii*	0.1243 (0.0129)	_	0.0488 (0.0092)
*P. gaisleri*	*P. kolombatovici* (Turkey‐Libya)	_	0.0538 (0.0073)	0.0186 (0.0052)
*P. gaisleri*	*P.* cf. *gaisleri*	0.0467 (0.0084)	_	0.0172 (0.0049)
*P. gaisleri*	*P. teneriffae*	_	_	0.0161 (0.0049)
*P. kolombatovici* (Balkans)	*P. christii*	0.1203 (0.0119)	_	0.0628 (0.0104)
*P. kolombatovici* (Balkans)	*P. kolombatovici* (Turkey‐Libya)	_	0.0492 (0.0067)	0.0131 (0.0043)
*P. kolombatovici* (Balkans)	*P.* cf. *gaisleri*	0.0573 (0.0093)	_	0.0241 (0.0060)
*P. kolombatovici* (Balkans)	*P. teneriffae*	_	_	0.0275 (0.0066)
*P. kolombatovici* (Turkey‐Libya)	*P. *cf. *gaisleri*	_	_	0.0197 (0.0050)
*P. kolombatovici* (Turkey‐Libya)	*P. teneriffae*	_	_	0.0228 (0.0059)

**Figure 5:**
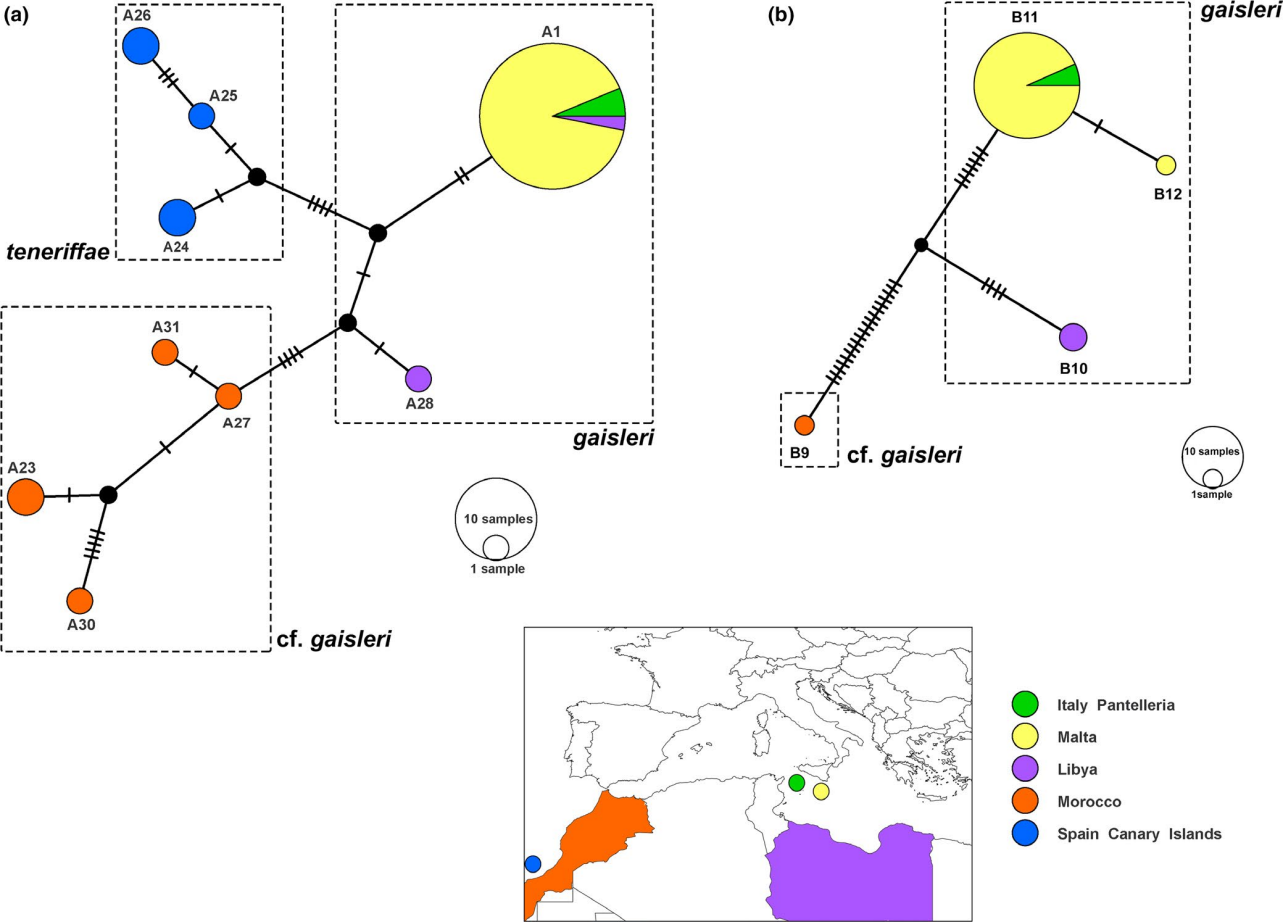
Median‐joining network of 16s rRNA (a) and COI (b) haplotypes of *Plecotus gaisleri*, *P. cf. gaisleri*, and *P. teneriffae* (see Table 1). Each circle represents a haplotype, and circle size is proportional to haplotype frequency. Colors indicate different sampling countries. Small black traits represent possible median vectors, while dashes represent substitutions

Based on haplotypes, *Plecotus* from Pantelleria shared the same sequences at all the three loci with Maltese *P. gaisleri* populations and also the same COI and 16s rRNA sequence with some Libyan *P. gaisleri.* Both Moroccan *P*. cf. *gaisleri* and *P. teneriffae* from the Canary Islands significantly differed from samples from Libya, Pantelleria, and Malta. Haplotype structure of COI and 16s rRNA regions is depicted in Figure 5 (A‐B; the haplotype network of ND1 is missing due to the lack of reference sequences for some taxa/populations belonging to the “*gaisleri”* complex).

## 4 DISCUSSION

In agreement with our hypothesis, we demonstrate that *P. gaisleri* occurs on the Italian island of Pantelleria, adding to the very recent confirmation that the species is present on Malta and Gozo (Batsleer et al., 2019; Mifsud & Vella, 2019). The occurrence on Pantelleria and the output of our SDM analysis make a strong case for a more generalized presence of this species at least on the islands that are scattered between the African and European coasts; thus, *P. gaisleri* should be fully regarded as part of the European bat fauna. Therefore, all official checklists for the continent and conservation directives and strategies should include this taxon. Our findings bring to seven the number of long‐eared bat species found in Europe, six of which are present in Italy, confirming the diversity hotspot role of the Italian Peninsula for mammals (Loy et al., 2019) and bats in particular.

The molecular screening conducted at the three loci suggests that the populations that are most closely related to the Italian one are those from Malta and, to a lower extent, Libya (Cyrenaica). Similarly, the two individuals caught on Pantelleria had body measurements that fall within the range known for African *P. gaisleri* (Benda et al., 2014) and are closer to those of individuals from Malta (Mifsud & Vella, 2018), which tend to be smaller than continental specimens.

Our modeling analysis suggests that the occurrence of *P. gaisleri* across its range is mainly driven by temperature of the coldest month: Lower temperatures may favor winter torpor in bats, when active foraging is not profitable, as indicated by the high importance of this bioclimatic variable in the potential distribution of Palearctic bats (e.g. Smeraldo et al., 2018). *Plecotus gaisleri* potential range is also characterized by relatively low values of isothermality, which indicates limited daily temperature variability in comparison with yearly variation. Isothermality is also considered among those variables directly affecting physiological performances of bats, as well as food or biomass availability (Ancillotto et al., 2018; Schoeman, Cotterill, Taylor, & Monadjem, 2013). Precipitation in the coldest quarter of the year also affected species distribution: in Mediterranean biomes, this factor may be interpreted as a proxy of water availability during the subsequent dry season. Land cover and elevation had a smaller influence on the potential occurrence of *P. gaisleri* across its range, yet according to our model areas with Mediterranean vegetation such as scrubland and dry forests, and complex mosaic landscapes were preferred, as well as altitudes <1,000 m a.s.l. These preferences are consistent with field observations that identify such habitats as important to foraging *P. gaisleri* (Benda & Aulagnier, 2013; Benda et al., 2014; Dalhoumi, Hedfi, Aissa, & Aulagnier, 2014, this study). The elevation limits correspond to those observed in N Africa (Tunisia), where the species occurs in both coastal and montane areas up to 950 m a.s.l (Benda & Aulagnier, 2013).

Our SDMs did well in estimating distribution of *P. gaisleri* on the islands between southern Italy and North Africa, as shown by validation of model performance. AUC values such as those we obtained (>0.98) are among the highest reported for published models (e.g., Moradi, Sheykhi Ilanloo, Kafash, & Yousefi, 2019; Smeraldo et al., 2018) and demonstrate a high predictive power of habitat suitability (Elith, Kearney, & Phillips, 2010). Our study was further supported by a high TSS value (e.g., Runquist, Lake, Tiffin, & Moeller, 2019; Smeraldo et al., 2020). Finally, all the presence records of *P. gaisleri* used for model validation fell in predicted suitable areas on both Pantelleria and Malta.

Our modeling exercise strongly supported *P. gaisleri* presence on the islands of the Sicily Channel and was successfully ground‐validated by our survey of Pantelleria and by the recent confirmed records from Malta and Gozo (Mifsud & Vella, 2019). All islands in the Sicily Channel provide suitable habitat for the species, yet records of long‐eared bats are only available from Malta, Gozo, and Pantelleria, that is, the largest ones, which were also those environmentally more similar to the continental range of *P. gaisleri*. *Plecotus* bats are effective colonizers of even remote or very small islands (e.g., *P. kolombatovici* on the Croatian island of Lokrum, 0.72 km^2^; Schofield et al.., 2018). The absence of records from other islands of the Sicily Channel may thus either reflect a genuine absence of *P. gaisleri* due to human pressure on such islands, or more likely insufficient surveying efforts. We therefore recommend that such islands are searched for the occurrence of this species based on the results of our modeling analysis.

Similarly, our models show that the entire island of Pantelleria provides suitable habitat for the species, but two coastal areas present environmental conditions that are especially close to those found in mainland Africa (Figures S1 and S2). In fact, our field records mainly refer to one of these areas, yet further efforts are needed to fully assess the species distribution on the island (Gastón & García‐Viñas, 2010).

As highlighted by Mifsud and Vella (2019), Mediterranean insular and Libyan *P. gaisleri* populations significantly differ from Moroccan *P.* cf. *gaisleri* as well as from *P. teneriffae* from the Canary Islands. This condition is also supported by the COI genetic distance values which are greater than the optimum threshold for species divergence of Palearctic echolocating bats (Galimberti et al., 2012). The Moroccan taxon may thus represent a new undescribed species, awaiting further sampling, multilocus genotyping, and formal description.

According to the new discoveries, *P. gaisleri* would be restricted to a very limited range in Europe (Batsleer et al., 2019; Mifsud & Vella, 2019; this work), even if accounting for the entire potential range in the islands across the Sicily Channel. We cannot rule out, however, that the species is present in other European areas such as Sicily, so this merits further investigation. Since the only records of *P. gaisleri* available for Europe are confined to islands*,* and refer to relatively small populations separated from the mainland, the entire European population is probably very small, fragmented, and isolated from other populations. Thus, the European population is potentially exposed to a high risk of extinction (Conenna et al., 2017). The high haplotype diversity we observed and the genetic differences from mainland Africa populations further highlight the importance of adopting special conservation measures to preserve such isolated populations.

Conservation of coastal areas is of fundamental importance for preserving bat populations on islands (Ancillotto, Rydell, Nardone, & Russo, 2014), particularly due to the high risk of anthropogenic disturbance in such fragile environments (Claudet & Fraschetti, 2010). For this reason, the conservation status of *P. gaisleri* in Europe is probably precarious, requiring special efforts to locate reproductive and wintering roosts, assess the species' spatial needs, and identify active and potential pressures to guarantee long‐term conservation.

Our work provides an example of how integrating field surveys, molecular analyses, and spatial modeling may help assess the presence of species at the edge of their known ranges, an important asset in conservation biology (Holt & Keitt, 2005; Razgour et al., 2016). This approach can also foster future research on the biogeography and taxonomy of cryptic species complexes such as that of Mediterranean long‐eared bats.

## CONFLICT OF INTEREST

None declared.

## AUTHOR CONTRIBUTIONS

**Leonardo Ancillotto:** Conceptualization (lead); formal analysis (lead); investigation (equal); writing–original draft (equal); writing–review and editing (equal). **Luciano Bosso:** Formal analysis (equal); investigation (equal); methodology (equal); software (lead); validation (lead); Writing–original draft (equal); writing–review and editing (equal). **Sonia Smeraldo:** Formal analysis (equal); software (equal). **Emiliano Mori:** Conceptualization (lead); formal analysis (equal); investigation (equal). **Giuseppe Mazza:** Investigation (equal). **Matthias Herkt:** Data curation (lead). **Andrea Galimberti:** Formal analysis (lead); Methodology (equal). **Fausto Ramazzotti:** Formal analysis (equal); methodology (equal). **Danilo Russo:** Conceptualization (lead); investigation (equal); supervision (lead); writing–original draft (equal); writing–review and editing (equal).

## Supporting information

Appendix S1

## Data Availability

Ecological raw data used in this study were obtained from literature and an online database (details in Materials and Methods). Sequenced data are available as part of database of the European Bioinformatics Institute of the European Molecular Biology Laboratory (EMBL‐EBI) with the accession numbers provided in Table 1.
