# Supplementary material for: An African bat in Europe, Plecotus gaisleri: Biogeographic and ecological insights from molecular taxonomy and Species Distribution Models
Source: Ecol Evol. 2020 Apr 29;10(12):5785–800. doi: 10.1002/ece3.6317 (PMC7319239; doi:10.1002/ece3.6317)
Supplement: Supplementary file 1 — Appendix S1 [file ECE3-10-5785-s001.docx]

**Supplementary Material**

**Table S1** Land cover categories used in the SDM analysis of *P. gaisleri* potential distribution

| Code | Land cover category |
| --- | --- |
| 0 | No data |
| 10 | Cropland, rainfed |
| 11 | Herbaceous cover |
| 12 | Tree or shrub cover |
| 20 | Cropland, irrigated or post-flooding |
| 30 | Mosaic cropland (>50%) / natural vegetation (tree, shrub, herbaceous cover) (<50%) |
| 40 | Mosaic natural vegetation (tree, shrub, herbaceous cover) (>50%) / cropland (<50%) |
| 50 | Tree cover, broadleaved, evergreen, closed to open (>15%) |
| 60 | Tree cover, broadleaved, deciduous, closed to open (>15%) |
| 61 | Tree cover, broadleaved, deciduous, closed (>40%) |
| 62 | Tree cover, broadleaved, deciduous, open (15-40%) |
| 70 | Tree cover, needleleaved, evergreen, closed to open (>15%) |
| 71 | Tree cover, needleleaved, evergreen, closed (>40%) |
| 72 | Tree cover, needleleaved, evergreen, open (15-40%) |
| 80 | Tree cover, needleleaved, deciduous, closed to open (>15%) |
| 81 | Tree cover, needleleaved, deciduous, closed (>40%) |
| 82 | Tree cover, needleleaved, deciduous, open (15-40%) |
| 90 | Tree cover, mixed leaf type (broadleaved and needleleaved) |
| 100 | Mosaic tree and shrub (>50%) / herbaceous cover (<50%) |
| 110 | Mosaic herbaceous cover (>50%) / tree and shrub (<50%) |
| 120 | Shrubland |
| 121 | Shrubland evergreen |
| 122 | Shrubland deciduous |
| 130 | Grassland |
| 140 | Lichens and mosses |
| 150 | Sparse vegetation (tree, shrub, herbaceous cover) (<15%) |
| 151 | Sparse tree (<15%) |
| 152 | Sparse shrub (<15%) |
| 153 | Sparse herbaceous cover (<15%) |
| 160 | Tree cover, flooded, fresh or brakish water |
| 170 | Tree cover, flooded, saline water |
| 180 | Shrub or herbaceous cover, flooded, fresh/saline/brakish water |
| 190 | Urban areas |
| 200 | Bare areas |
| 201 | Consolidated bare areas |
| 202 | Unconsolidated bare areas |
| 210 | Water bodies |
| 220 | Permanent snow and ice |

| **Island** | **Accuracy** | **Source** | **Year** | **Comment** | **Logistic value** | **Binary value** |
| --- | --- | --- | --- | --- | --- | --- |
| Pantelleria | <1km | This study | 2019 | Roost | 0.6 | 1 |
| Pantelleria | <1km | This study | 2019 | Acoustic | 0.7 | 1 |
| Pantelleria | <1km | This study | 2019 | Acoustic | 0.9 | 1 |
| Pantelleria | <1km | This study | 2019 | Acoustic | 0.7 | 1 |
| Pantelleria | <1km | This study | 2019 | Acoustic | 0.7 | 1 |
| Malta | <5km | MEPA - Malta Environment & Planning Authority (2010). Available at https://www.eurobats.org/sites/default/files/documents/pdf/National_Reports/nat_rep_Malta_2010.pdf | 2010 | Not specified | 0.7 | 1 |
| Malta | <5km | MEPA - Malta Environment & Planning Authority (2010). Available at https://www.eurobats.org/sites/default/files/documents/pdf/National_Reports/nat_rep_Malta_2010.pdf | 2010 | Not specified | 0.7 | 1 |
| Malta | <5km | MEPA - Malta Environment & Planning Authority (2010). Available at https://www.eurobats.org/sites/default/files/documents/pdf/National_Reports/nat_rep_Malta_2010.pdf | 2010 | Not specified | 0.7 | 1 |
| Malta | <5km | MEPA - Malta Environment & Planning Authority (2010). Available at https://www.eurobats.org/sites/default/files/documents/pdf/National_Reports/nat_rep_Malta_2010.pdf | 2010 | Not specified | 0.7 | 1 |
| Malta | <5km | Batsleer, F., Portelli, E., Borg, J. J., Kiefer, A., Veith, M., & Dekeuleire, D. (2019). Maltese bats show phylogeographic affiliation with North-Africa: implications for conservation. *Hystrix* 30, 172-177. | 2019 | Molecular identification | 0.7 | 1 |
| Malta | <1km | Borg, J. J., & Sammut, P. M. (2002). Note on the diet of a Grey Long-eared Bat, Plecotus austriacus (Fischer, 1829) from Mdina. Malta (Chiroptera, Vespertilionidae). The Central Mediterranean Naturalist, 3, 171-172 | 2001 | Roost | 0.7 | 1 |
| Pantelleria | <1km | Zava, B., & Lo Valvo, F. (1990). First record of *Suncus etruscus* and notes on the bats of Pantelleria island, Italy. *Mammalia*, *54*(4), 661-663. | 1990 | Roost | 0.7 | 1 |

**Table S2** Presence records of *P. gaisleri* used in model validation


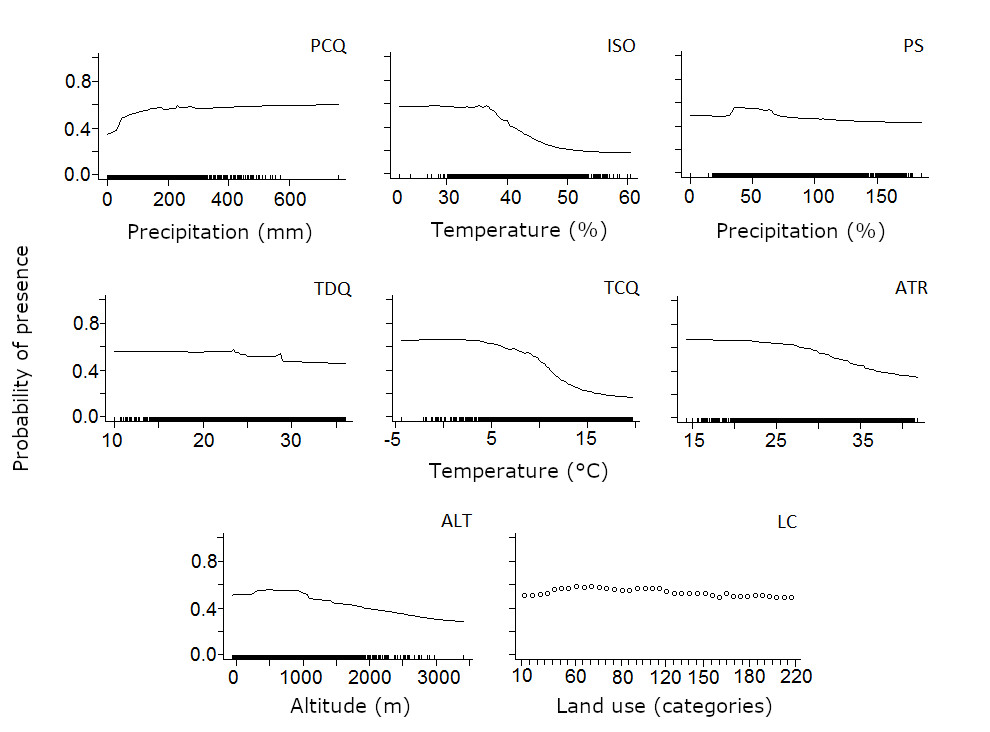


**Figure S1** Response curves for the eight variables used to model *Plecotus gaisleri* potential distribution in Africa obtained with the “biomod2” computer platform. Response curves correlate probability of occurrence (y axis) with values of the explanatory variables (x axis). Each curve represents one variable (PCQ = precipitation of coldest quarter; ISO = isothermality; PS = precipitation seasonality; TDQ = mean temperature of driest quarter; TCQ = mean temperature of coldest quarter; ATR = annual temperature range; ALT = altitude; LC= land cover (for further details regarding the categories to see Table S1). Distances are expressed in decimal degrees, precipitation in mm/% and temperature in °C


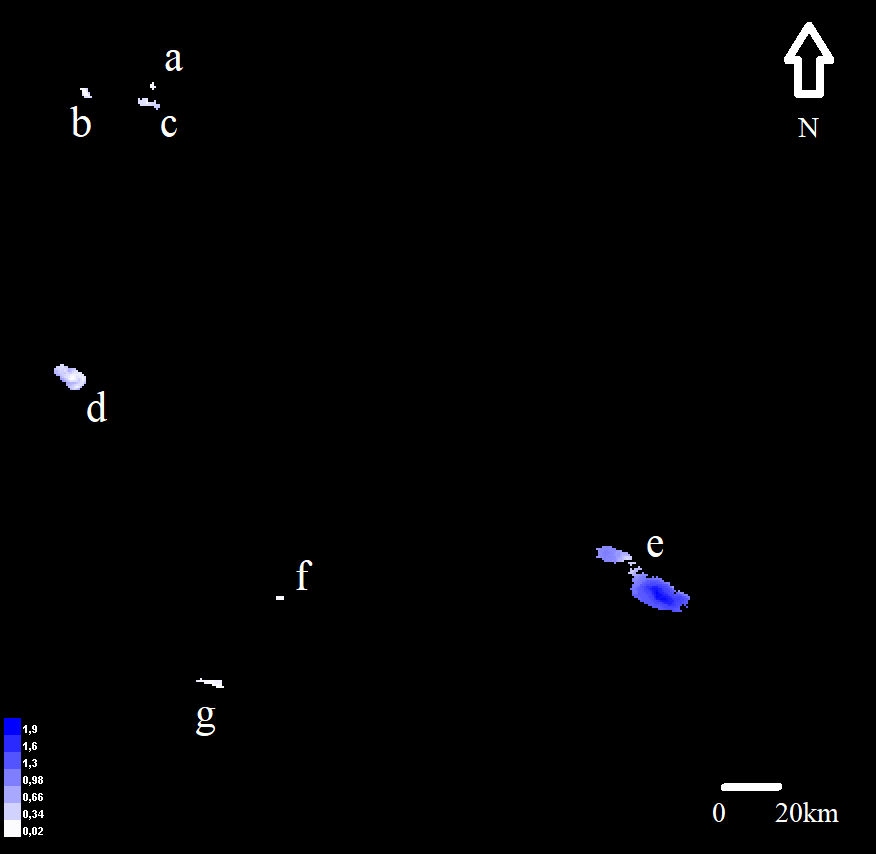


**Figure S2** MESS map of *P. gaisleri* in the projection areas. The islands are located as follows: Levanzo (a), Marettimo (b), Favignana (c), Pantelleria (d), Malta and Gozo (e), Linosa (f) e Lampedusa (g). The map shows similarity of the environmental variables between training and projection data. Environmental similarity ranged from negative (red) to positive values (blue) through zero (white). In this picture, areas in blue or close to blue have one or more environmental variables whose values fall within the range observed in the training area (Morocco, Algeria, Tunisia and Libya)
